# Gene expression profiles of white bass (*Morone chrysops*) and hybrid striped bass (*M. chrysops* x *M. saxatilis*) gill tissue following *Flavobacterium covae* infection

**DOI:** 10.1016/j.cirep.2024.200144

**Published:** 2024-04-19

**Authors:** Linnea K. Andersen, Jason W. Abernathy, Bradley D. Farmer, Miles D. Lange, Matthew E. McEntire, Steven D. Rawles

**Affiliations:** a Aquatic Animal Health Research Unit (AAHRU), Agricultural Research Service, United States Department of Agriculture, Auburn, AL, USA; b Harry K. Dupree Stuttgart National Aquaculture Research Center (HKDSNARC), Agricultural Research Service, United States Department of Agriculture, Stuttgart, AR, USA

**Keywords:** White bass, Hybrid striped bass, Moronid, Morone, Flavobacterium columnare, Flavobacterium covae, Mucosal immune response, Transcriptome, RNA sequencing

## Abstract

Columnaris disease is a prevalent disease in freshwater environments caused by the ubiquitous aquatic pathogen *Flavobacterium* species*.* Adhesion to the external mucosal surfaces of fishes is the initial stage of infection, and the gills specifically have been identified as both a primary target and release site for this pathogen. Demonstrated here and in previous research, the hybrid striped bass (*Morone chrysops* x *M. saxatilis*), a prominent United States aquaculture product, is more susceptible to infection with *F. covae* than the maternal white bass (*M. chrysops*) parental species. To further elucidate the mechanisms underlying differences in resistance between these fish we examined gill gene expression profiles using RNA sequencing at different timepoints after *F. covae* infection. Patterns of differential gene expression and association with key enrichment terms indicate the effective immune response observed in white bass includes the up-regulation of multiple cytokines (IL-1β, IL-17C, TNF-α, G-CSF, IL-8, CCL16, CXCL9), hepcidins (HAMP and HAMP2), ribosomal subunit components (e.g., RPL13), and, interestingly, the down-regulation of leptin (LEPA) during initial (1 – 4 h) stages of infection. Conversely, up-regulation of the same genes was not detected in hybrid striped bass until 24 h after infection, indicating a delay in immune response mechanisms that is ultimately ineffective in protecting the host, as this was concurrent with the onset of mortality in these fish. Collectively, the presented results include several putative pathways and candidate genes for further investigation toward characterizing immune defense mechanisms underlying the resistance (white bass) and susceptibility (hybrid striped bass) for selective breeding efforts and/or biotechnological intervention.

## Highlights

• White bass exhibit greater resistance to *F. covae* than hybrid striped bass.
• *F. covae* elicits a cytokine-mediated response in white and hybrid striped bass gill.
• White bass immune response is mounted earlier than the hybrid in response to *F. covae*.

## Introduction

Columnaris disease is a prevalent disease that impacts numerous wild and cultured freshwater fishes worldwide and leads to tens of millions in economic losses throughout the global aquaculture industry each year [1]. The causative agents of columnaris are the ubiquitous and opportunistic gram-negative bacteria previously referred to collectively as *Flavobacterium columnare* that have since been understood to represent four phylogenetically distinct groups that vary in virulence between host species: *F. columnare* (Group 1), *F. covae* (Group 2), *F. davisii* (Group 3), and *F. oreochromis* (Group 4) [2,3]. As with many aquatic pathogens, *Flavobacterium* spp. infection generally begins via adhesion to the mucosal surfaces (skin, gills, intestine), which are the first physical and biochemical (immunological) barrier between a host organism and the external environment [4]. The gill has been identified as the primary attachment site of *Flavobacterium* spp. and transmission can occur indirectly if present in the water column and fish-to-fish through cohabitation or direct contact with infected fish, both active and post-mortem, or observably healthy fish acting as carriers from a past infection [5]. The clinical presentation of columnaris ranges from visible gill lesions and severe tissue damage to mortality prior to the development of external markers of infection depending on the strain, water quality parameters, and age of host, in addition to other factors [5].

Hybrid striped bass (HSB) are a prominent aquaculture product in the United States (fourth largest by farm gate value), with the “reciprocal”, or “sunshine,” cross of white bass (WB, *Morone chrysops*) females with striped bass (SB, *M. saxatilis*) males being the most commonly produced [6]. The cross between freshwater WB and euryhaline SB exhibits hybrid vigor (heterosis) in many agriculturally-important traits, such as tolerance to various water quality parameters, feeding behavior, metabolism (bioenergetics), and response to handling stress [7]. In disease resistance, however, HSB do not invariably exhibit the same hybrid vigor across pathogens and environmental conditions [7,8]. Previous studies of *F. covae* infection in WB, SB, and their hybrid have found that WB demonstrate the greatest resistance, SB the greatest susceptibility, and HSB intermediate, whereby the incidence of mortality is higher and occurs more rapidly than in WB although not as rapidly as observed in SB [7,8]. Farmer et al. [8] also demonstrated that multiple concentrations (1–100 µg/mL) of SB mucus facilitate *F. covae* bacterial growth and biofilm formation to a greater extent than WB and HSB mucus, which displayed similar trends of suppression and stimulation across concentrations. Further, gill histopathology conducted previously by Fuller et al. [7] 24 h after *F. covae* challenge showed severe gill damage (lesions, necrosis, hyperplasia) in HSB compared to WB and the unchallenged controls.

The current study examines gill gene expression in WB and HSB at three timepoints throughout *F. covae* infection to gain insight into the cellular processes that underlie the observed variation in resistance between these fish. The work described herein also led to the identification of putative targets for SB selective breeding studies, as the differences in columnaris susceptibility suggest potential to focus efforts on cultivating lines with greater resistance akin to what is observed in the WB parent.

## Materials and methods

### Ethics statement

Animal care and experimental protocols were approved by the Harry K. Dupree Stuttgart National Aquaculture Research Center (HKDSNARC) Institutional Animal Care and Use Committee (IACUC) and conformed to USDA Agricultural Research Service Policies and Procedures 130.4 and 635.1.

### Experimental animals and bacteria challenge

A total of 480 fingerling WB (14.3 *g* ± 2.4 g; 10.6 cm ± 0.4 cm) and HSB (22.97 *g* ± 4.6 g; 12.7 cm ± 0.9 cm) were randomly selected from populations spawned indoors and reared in ponds to 2–3 months of age at the Harry K. Dupree Stuttgart National Aquaculture Research Center (Stuttgart, Arkansas, USA) and stocked into 16 replicate 18 L aquaria (8 for WB and 8 for HSB; *n* = 30 fish per tank). Fish were fed to apparent satiation 3 days per week, for 2 weeks prior to the study to acclimate. Fish were not fed on the day before bacterial challenge or thereafter.

For the *F. covae* challenge, tanks used for sampling were assigned randomly prior to the start of the experiment. Fish sampled at each time point were arbitrarily selected from the randomly designated sample tanks. WB and HSB in the control group (0 h) were first given a mock treatment of 10 mL sterile culture media and gill tissues were sampled from WB and HSB individuals (*n* = 6 WB and *n* = 6 HSB). The remaining fish were challenged by the addition of 10 mL culture of *F. covae* isolate LSU-066–04 [9] (0.70 absorbance at 550 nm; 6.3 × 10^^^6 CFU/mL) to each aquaria. Water was provided through an ultra-low-flow water delivery system at a temperature of 21–22ºC and a flow rate of 30 mL/min for 24 h and then flow was increased to 200 mL/min for the duration of the study [8,10]. Gill tissues were sampled from fish at three timepoints after the challenge had been initiated, at 1-hour post-exposure (1 h), 4 h post-exposure (4 h), and 24 h post-exposure (24 h) (*n* = 6 WB and *n* = 6 HSB at each timepoint; *N* = 48 individuals sampled total including controls). Fish in aquaria not used for sampling were observed for 8 days and survival data was analyzed using Kaplan-Meier log rank survival analysis followed by Holms-Sidak post-hoc analysis features (alpha=0.05; SigmaPlot v.13, Systat Software Inc., San Jose, CA.). At the end of the 8 day study, a random sampling of control fish were confirmed to be pathogen-free. Challenged fish were confirmed to be infected with *F. covae* and exhibited clinical signs consistent with the infection model. Remaining fish were euthanized with tricaine methanesulfonate (MS-222; Sigma-Aldrich, St. Louis, MO, USA) at 300 mg/L buffered with sodium bicarbonate at the conclusion of the study (Day 8).

### RNA extraction, library preparation, and sequencing

At each indicated time-point in the experimental challenge, the left second gill arch from each fish was excised, immediately submerged in RNA*later*™ (Life Technologies, CA, USA) and stored at −80.0 °C prior to use. Total RNA was extracted from the gill filaments with the Qiagen RNeasy Mini Kit (Qiagen Inc., Hilden, Germany) per the manufacturer's protocol. Total RNA was treated with Amplification Grade DNase I (Sigma-Aldrich, St. Louis, MO, USA) and then ethanol-precipitated prior to library construction. RNA quantity was assessed using a spectrophotometer (NanoDrop 1000, ThermoFisher Scientific, USA). RNA quality was assessed using the Agilent RNA 6000 Nano Kit on the 2100 BioAnalyzer instrument (Santa Clara, CA). All total RNA samples had RNA Integrity Numbers (RIN) greater than eight.

Each sample of total RNA was standardized to 100 ng and then proceeded to create RNA sequencing libraries using the NEBNext Ultra II Directional RNA Library Prep Kit for Illumina (New England Biolabs, Ipswich, MA) with the NEBNext Poly(A) mRNA Magnetic Isolation Module (New England Biolabs). Barcodes used for multiplexing were the NEBNext Multiplex Oligos for Illumina, Index Primers Sets 1 and 2. Libraries were quantified using the NEBNext Library Quant Kit for Illumina (New England Biolabs) on a Roche LightCycler 96 instrument (Indianapolis, IN), normalized to 5 nM and pooled. Pooled libraries were sent to a service provider (Macrogen Corp., Rockville, MD, USA) for high-throughput RNA sequencing (RNA-Seq) on an Illumina HiSeq X (San Diego, CA) in a 2 × 150 bp paired-end configuration. Base-calling and demultiplexing were performed using the native Illumina software and packages RTA (Real Time Analysis) and bcl2fastq by the sequencing service provider and raw FASTQ files provided for downstream analyses.

### Creation of reference white bass protein-coding transcriptome assembly

A total of 185,531 published white bass transcriptome contigs from the transcriptome shotgun assembly GAZY00000000.1 [11] were re-analyzed and a curated set of protein-coding transcripts selected to detect differentially expressed genes (DEGs), herein referred to as white bass transcriptome. To create the WB transcriptome, all transcripts were analyzed by BLASTx to the non-redundant (*nr*) database and only those with blast score E-value < e-20 were retained. Then, several measures were taken to reduce redundancy. First, contigs with > 90 % similarity were clustered using CD-HIT-EST [12]. Sequence similarity was further detected using BLAT [13], where duplicates at *a* > 80 % Similarity threshold were removed. Then, custom shell scripts were used to manually curate these clusters, where those with top-hit BLASTx returns of hypothetical/unknown protein identities were removed, and those with duplicate top-hit descriptions were reduced to include only the longest sequence. This produced a protein-coding WB transcriptome containing 20,914 sequences. BUSCO analysis [14] against the Actinopterygii_odb9 lineage dataset indicated a substantially completed transcriptome, with only 8.4 % reported as missing.

### Bioinformatics

Starting with raw FASTQ files, RNA-Seq data were processed for quality control (QC) using FastQC (v.0.11.8) [15] and Trimmomatic (v.0.38) [16]. Default parameters were used for the removal of Illumina adapters and trimming of low-quality reads (Q25) and reads below 15 bp in length. Overrepresentation of 18S, 5.8S, and 28S ribosomal RNA (rRNA) sequences were identified in two samples, one WB and one HSB collected at 4 h post-infection (identified as WB-1-t4hr and HB-4-t4hr, respectively). These individuals were dropped from subsequent analyses and reads generated from remaining individuals were filtered for potential contamination via alignment to the *M. saxatilis* 18S, 5.8S, and 28S sequences available in GenBank (Accessions: XR_004797324.1, XR_004797323.1, and XR_004797325.1, respectively) using the default parameters for the STAR Gapped Mapper (v.2.7.8a) [17] and Samtools (v.1.12) [18]. The subsequent unaligned reads were then aligned to the WB transcriptome described above using the same pipeline and default parameters. Read-counts were generated from the resulting BAM files using default parameters of HTSeq (v.0.9.0) [19]. Pairwise gene expression fold-changes from both WB and HSB at each time post-infection (1 h, 4 h, and 24 h) relative to the respective WB or HSB 0 h control group were estimated with the edgeR (v.3.28.0) package of R-bioconductor [20] using the Weighted Trimmed mean of M-values (TMM) normalization method, Exact Test, and “robust” statistical filter. A p-value less than 5 % after adjusting for multiple testing (adj-*p* < 0.05) as well as log fold-change values greater than one (logFC > 1) and less than negative one (logFC < - 1) were set as thresholds to determine significant up- and down-regulation, respectively. An Edwards-Venn diagram was created using jvenn to visualize overlapping DEGs between the six comparisons [21]. The Fisher's Exact Test and Gene Set Enrichment Analysis (GSEA) were used to perform Gene Ontology (GO) enrichment analyses. The Fisher's Exact Test was applied using default parameters of the FatiGO package (filter via adjusted p-value of *p* < 0.05, Benjamini-Hochberg FDR used for multiple test correction) to determine if up- and down-regulated genes from each pairwise comparison were over- and/or under-represented (two-tailed test) relative to the WB transcriptome [22]. Enrichment of all major GO categories (Biological Processes, BP, Molecular Function, MF, and Cellular Component, CC) was determined via GSEA using differentially expressed genes (DEGs) from each pairwise comparisons ordered by increasing FDR-adjusted p-value < 0.05 [23].

### RNA sequencing validation (RT-qPCR)

Nine genes of interest (GOI) were selected for validation of RNA-seq data by reverse-transcription quantitative PCR (RT-qPCR): six GOIs to assess each of the sample timepoints and three immune-related GOIs. The selected GOIs are listed here and alongside gene-specific primer information in **Supplemental Table 1**: osmotic stress transcription factor 1 (OSTF1, or TSC22D3); proto-oncogene c-Fos-like (FOS); sequestosome-1 isoform X2 (SQSTM1); C2 domain-containing protein 2-like isoform X1 (C2CD2); hepcidin (HAMP); interleukin-1 beta (IL-1β); interleukin-8 (IL-8, or CXCL8); ubiquitin-40S ribosomal protein S27a (RPS27A); keratin, type II cytoskeletal 8-like (KRT8); and housekeeping gene TATA-box-binding protein (TBP). Primers were designed using NCBI Primer-BLAST [24]. Primer sets were also aligned to the WB genome sequence (NCBI: GCA_019097615.1, DOM_MoChry_2.0) prior to selection for validation.

Total RNA isolates used to generate RNA-seq libraries from all HSB and WB samples were used for RT-qPCR validation. RNA sample quality and quantity was re-assessed via spectrophotometry (260/280 was 2.019 ± 0.072, minimum was 1.89) using a BioTek EPOCH2 microplate reader (BioTek Instruments, Winooski, VT) and automated electrophoresis (RINe value was 9.61 ± 0.49, minimum was 8.10) using an Agilent TapeStation 4200 with RNA ScreenTape (Agilent Technologies, Santa Clara, CA, USA). Each sample was normalized to 200 ng/μL with nuclease-free water and then cDNA was synthesized using the NEB LunaScript® RT SuperMix Kit (Ipswich, MA) according to the manufacturer protocol including the preparation of the no-RT and no template controls. Resulting cDNA and controls were diluted to 2 ng/μL with nuclease-free water and stored at −20 °C until RT-qPCR. The NEB Luna® Universal qPCR Master Mix kit (Ipswich, MA) was used for RT-qPCR according to manufacturer instructions with the exception that the final reaction volume was scaled-down to 10 μL per well (5 μL Luna Universal qPCR mix, 0.5 μL forward primer (1 μM), 0.5 μL reverse primer (1 μM), 2 μL cDNA or control, and 2 μL nuclease-free water). Technical replicates were performed in triplicate for each sample in a 384-well format. The RT-qPCR assays were performed using the Roche LightCycler 480 Instrument II (Roche Diagnostics, Indianapolis, IN) and analyzed with the corresponding software (LCS480) that allowed for the collection of cycle threshold (Ct) values. Relative quantification was calculated by normalizing the geometric average of the reference genes to the housekeeping gene using the 2^−ΔΔCT^ method [25,26].

## Results

### Survival of bass after Flavobacterium covae challenge

**Fig. 1:**
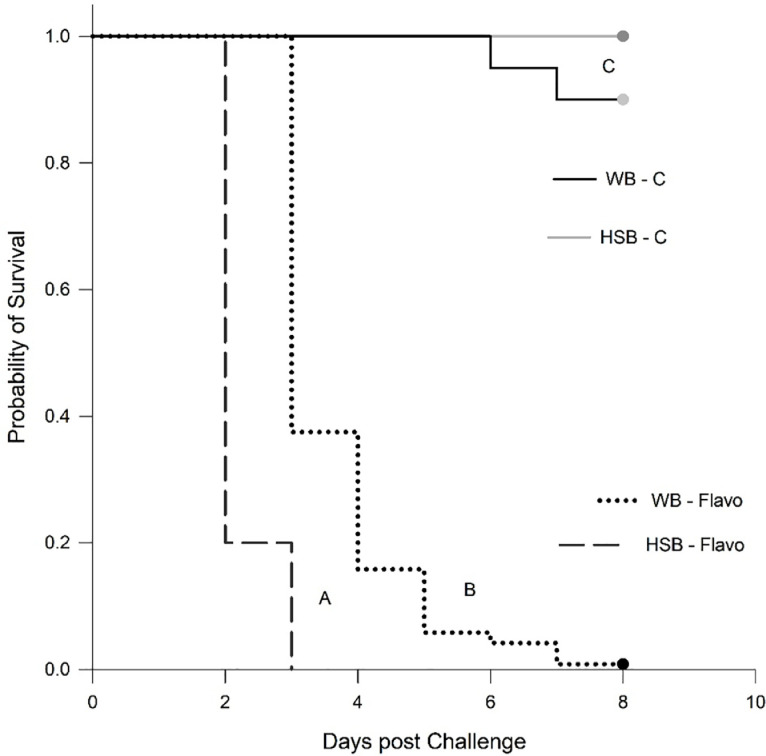
Kaplan-Meier survival analysis of white bass (WB - Flavo) and hybrid striped bass (HSB - Flavo) challenged with *Flavobacterium covae* isolate LSU-066–04 as well as non-challenged control fish (WB - C and HSB - C). Different letters next to each curve indicate significant differences at *p* < 0.05.

The challenge experiment duration was 8 days, with mortalities starting 48 h after challenge in HSB and 72 h for WB. All fish were alive during the tissue sampling (0–24 h). One hundred percent mortality was observed on day 3 for HSB with WB reaching 96 % by the conclusion of the study on day 8 (Fig. 1). The survival data showed significant differences (*p* = 0.001) between the bass species as well as significant differences between challenged fish and unchallenged control fish (*p* = 0.001). The disease progressed as expected and clinical signs included fin erosion, areas of necrosis on the gills and necrotic lesions on the skin and fins [5,7,8].

### RNA sequencing and validation

A total of 48 RNA-seq libraries with an average sequencing depth of 21.0 M paired-end reads per sample were generated that was reduced to ∼20 M paired-end reads per sample after QC and trimming. Sequencing reads for two individuals sampled at 4 h, one WB and one HSB, were dropped from analysis due to overrepresentation of 18S, 5.8S, and 28S rRNA sequences (52.61 % and 68.99 % alignment to these sequences, respectively). The mean alignment of the remaining samples (*n* = 46) to these rRNA sequences was 3.17 % ± 0.044 %. The rRNA-filtered reads had an overall mapping rate of 61.65 % to the WB transcriptome, with HSB having a mean mapping rate of 58.79 % and WB 64.51 %. The directionality (up-/down-regulation) and magnitude of gene expression determined via RT-qPCR assay validated the gene expression patterns identified via RNA-Seq analysis (**Supplemental Table 1**).

### Differential gene expression

**Fig. 2:**
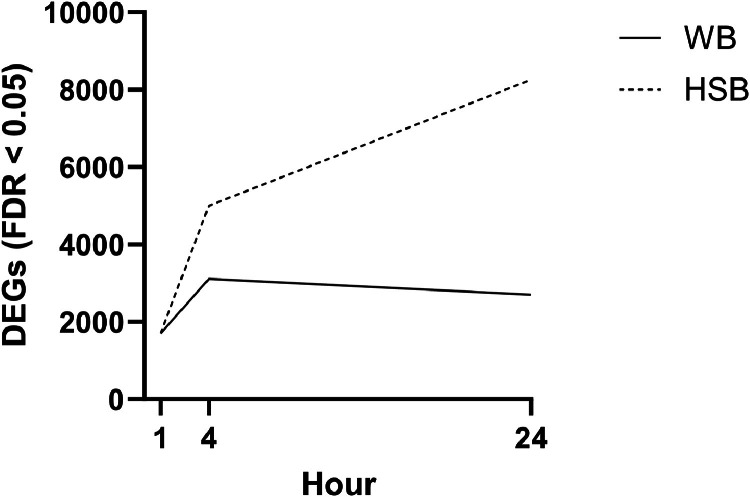
The number of differentially expressed genes (DEGs, false discovery rate, FDR, adjusted p-values < 0.05) in gill tissue of white bass (WB) and hybrid striped bass (HSB) at timepoints 1 h, 4 h, and 24 h post-infection with *Flavobacterium covae* determined via pairwise comparisons to the species-specific 0 h control condition.

**Fig. 3:**
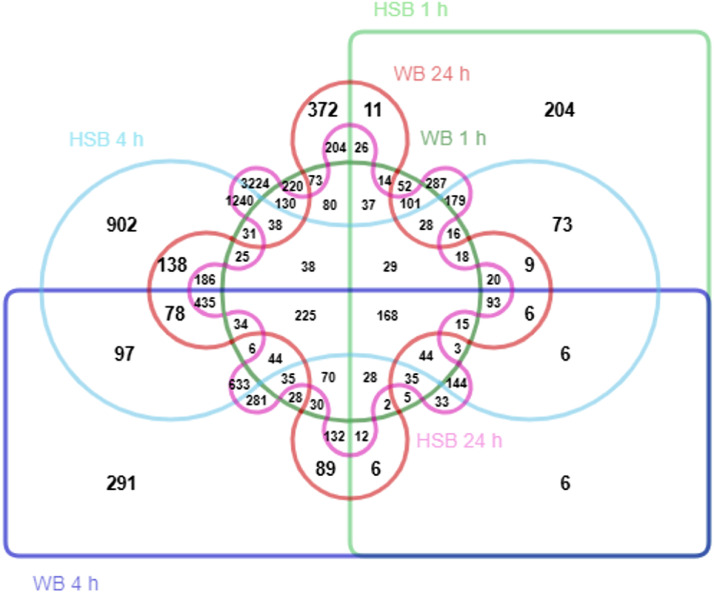
Edwards-Venn diagram of differentially expressed genes (DEGs, false discovery rate, FDR, adjusted p-values < 0.05) in gill tissue of white bass (WB) and hybrid striped bass (HSB) at each timepoint after infection (1 h, 4 h, and 24 h) with *Flavobacterium covae* relative to the species-specific 0 h control (e.g., “HSB 24 h” are DEGs identified through a pairwise comparison of gene expression in HSB at 24 h post-infection with *F. covae* relative to 0 h prior to exposure).

A total of 11,119 unique transcripts were differentially expressed in at least one comparison and 9,332 of these transcripts aligned with a sequence in the WB transcriptome that had been annotated to the gene-name level. Table 1 shows the number of differentially expressed genes (DEGs, FDR adjusted p-value < 0.05) and up- and down-regulated DEGs at a set fold-change cutoff (logFC > 1.0 or < −1.0, respectively) identified from pairwise comparisons of WB and HSB at each timepoint relative to the respective 0 h control group. In WB, the number of DEGs relative to the control condition (0 h) increased between 1 h and 4 h and decreased at 24 h from 4 h, whereas in HSB the number of DEGs relative to the control condition continued to increase through 24 h (Table 1**,** Fig. 2). Fig. 3 shows the number of DEGs shared between each comparison, a total of 168 genes were identified as differentially expressed between all comparisons. In comparisons of WB and HSB DEGs identified at the same time-points, 595 DEGs were shared between those identified for WB and HSB at 1 h relative to the species-specific 0 h control conditions (34.96 % of 1,702 DEGs for WB and 34.80 % of 1,710 for HSB), 23 of which are up-regulated and 6 down-regulated in both groups. At 4 h post-infection, 2,031 of the WB and HSB DEGs were shared (65.22 % of 3,114 DEGs for WB and 40.61 % of 5,001 for HSB), 114 of which are up- and 91 are down-regulated in both groups. At 24 h, 1,783 of the WB and HSB DEGs are shared (65.96 % of 2,703 DEGs for WB and 21.59 % of 8,259 for HSB), 133 of which are up- and 61 are down-regulated in both groups. All gene transcripts alongside FDR and logFC values for the corresponding comparison are provided in **Supplemental Table 2**.

**Table 1:** Differentially expressed genes (DEGs) in gill tissues of white bass (WB) and hybrid striped bass (HSB) after infection with *Flavobacterium covae* determined via pairwise comparisons of WB and HSB at each hour post-infection (“Time (h)”, 1 h, 4 h, and 24 h) relative to the species-specific 0 h control condition (i.e., prior to exposure). Reported values are number of DEGs with false discovery rate (FDR) adjusted p-values < 0.05 followed by the number of DEGs with FDR < 0.01 in parentheses. Up- and down-regulated DEGs are those with a log fold-change (logFC) value of > 1.0 or < - 1.0, respectively.

**WB**			
**Time (h)**	**Total DEGs**	**Up-Regulated DEGs**	**Down-Regulated DEGs**
1	1702 (747)	107	75
4	3114 (1476)	364	504
24	2703 (1180)	303	199

**HSB**			
**Time (h)**	**Total DEGs**	**Up-Regulated DEGs**	**Down-Regulated DEGs**
1	1710 (893)	130	88
4	5001 (3153)	362	386
24	8259 (5941)	2113	1527

### Gene ontology enrichment analyses

Gene ontology (GO) enrichment analyses of up- and down-regulated (Fisher's Exact Test) and all (GSEA) DEGs detected in pairwise comparisons of WB and HSB 1 h, 4 h, and 24 h after *F. covae* infection and the respective 0 h control groups identified a total of 681 unique GO terms enriched in at least one comparison. Of the unique GO terms, 199 were identified through both enrichment analysis approaches. Specific Fisher's Exact Test and GSEA results are reported (Sections 3.4.1 and 3.4.2, respectively) and integrated in the context of immune processes (Section 3.5) in the subsequent sections.

#### Representation of GO terms via Fisher's exact test

Overrepresented and/or underrepresented GO terms were detected via Fisher's Exact Test among gene transcripts up-regulated in WB at 1 h (29 GO terms; 16 BP, 2 MF, 11 CC), 4 h (12 GO terms; 5 BP, 6 MF, 1 CC), 24 h (33 GO terms; 11 BP, 2 MF, 20 CC), and HSB 24 h (332 GO terms; 210 BP, 54 MF, 68 CC) and those down-regulated in WB at 4 h (9 GO terms; 2 BP, 7 MF) and HSB at 4 h (3 GO terms; 2 MF, 1 CC) and 24 h (66 GO terms; 33 BP, 19 MF, 14 CC) after *F. covae* infection relative to the respective 0 h control groups and the WB transcriptome as reference. All overrepresented GO terms (381 unique terms total) are listed in **Supplemental Table 3**.

#### Gene set enrichment analysis (GSEA)

Among the GO terms identified as enriched for WB at 1 h (147 terms), 4 h (204 terms), and 24 h (214 terms) after *F. covae* infection, 293 were unique (153 BP, 77 MF, 63 CC) and 272 terms were repeated between at least two of the three comparisons. Among the GO terms identified as enriched for HSB at 1 h (44 terms), 4 h (144 terms), and 24 h (397 terms) after *F. covae* infection, 423 were unique (256 BP, 100 MF, 67 CC) and 162 terms were repeated. In all, 499 unique (of 1,150 total) GO terms were identified between the WB and HSB GSEA results and are listed in **Supplemental Table 4**. Of these, 76 were only identified among one or more WB comparisons, 206 among one or more HSB comparisons, and 217 were shared between WB and HSB comparisons (Table 2).

**Table 2:** Unique and shared GO Categories (FDR q-value < 0.05) determined through comparisons of gill gene expression Gene Set Enrichment Analysis (GSEA) between white bass (WB) and hybrid striped bass (HSB) after infection with *Flavobacterium covae* (1 h, 4 h, and 24 h post-infection) relative to the 0 h control (i.e., prior to exposure). Total values shown do not reflect duplicate GO terms present within WB or HSB comparison lists.

GO Category	Unique to WB	Unique to HSB	Total Shared
Biological Processes (BP)	41	144	112
Molecular Functions (MF)	24	47	53
Cellular Component (CC)	11	15	52

Seventeen (17) of the 29 terms identified as overrepresented in WB 1 h post-infection via Fisher's Exact Test were also identified as enriched through GSEA in WB 1 h post-infection as well as most or all other comparisons. Similarly, 9 of the 21 terms identified for WB 4 h post-infection and 27 of the 33 identified for WB 24 h post-infection via Fisher's Exact Test were also identified in the same and other comparisons through GSEA. Two hundred and fourteen (214) of the 398 terms identified for HSB 24 h post-infection via Fisher's Exact Test were also identified as enriched through GSEA, although four of these were not also identified as enriched in the same comparison via GSEA.

### Enrichment and differential expression patterns of immune processes

Enrichment analyses identified several GO terms directly related to immune system processes in WB and HSB after *F. covae* infection, such as cytokine activity, pathogen/antigen recognition, and response to stress (Table 3). Table 4 lists GOIs associated with the GO terms in Table 3 and/or other immune functions.

**Table 3:** Select GO terms broadly related to immune system function and regulation determined via analyses of up- and down-regulated (Fisher's Exact Test) and/or all (Gene Set Enrichment Analysis, GSEA) DEGs detected in pairwise comparisons of WB and HSB 1 h, 4 h, and 24 h after *Flavobacterium covae* infection relative to species-specific 0 h controls. Comparisons are indicated as species (WB/HSB) and time after infection. All listed terms detected by Fisher's Exact Test were overrepresented among up-regulated genes for a given comparison and the reference transcriptome. The associated the associated adjusted p-value is provided under “Fisher's”. All listed terms detected by GSEA were associated with DEGs of greater significance (i.e., smaller FDR p-value) that accumulated towards the top of the input list. The Normalized Enrichment Scores (NES) describing the extent of DEG overrepresentation at the top, and where higher values indicate less randomness, are provided under “GSEA”. Dashes “-” indicate a given GO term was not identified by a given test.

GO Term	Comparison	Fisher's	GSEA
antioxidant activity (GO:0016209)	WB 24 h	–	1.893
blood coagulation (GO:0007596)	HSB 24 h	–	1.577
canonical NF-kappaB signal transduction (GO:0007249)	WB 24 h	–	1.692
cell chemotaxis (GO:0060326)	HSB 24 h	5.78E-05	1.660
cell redox homeostasis (GO:0045454)	WB 4 h	–	1.784
	HSB 24 h	–	1.780
cellular detoxification (GO:1990748)	WB 24 h	–	1.906
cellular response to cytokine stimulus (GO:0071345)	WB 1 h	–	1.761
chemokine activity (GO:0008009)	WB 1 h	–	1.747
	WB 24 h	–	1.757
	HSB 24 h	4.31E-06	–
chemokine receptor binding (GO:0042379)	WB 1 h	–	1.738
	WB 24 h	–	1.766
	HSB 24 h	7.26E-06	–
chemotaxis (GO:0006935)	HSB 24 h	2.56E-03	–
cytokine activity (GO:0005125)	WB 1 h	–	1.858
	HSB 24 h	2.73E-11	1.858
cytokine-mediated signaling pathway (GO:0019221)	WB 1 h	–	1.699
cytokine receptor binding (GO:0005126)	HSB 24 h	3.63E-05	–
defense response (GO:0006952)	HSB 24 h	3.01E-04	1.556
detection of external stimulus (GO:0009581)	WB 4 h	–	1.672
detoxification (GO:0098754)	WB 24 h	–	1.774
immune response (GO:0006955)	WB 1 h	–	1.764
	HSB 24 h	9.91E-04	–
immune system process (GO:0002376)	HSB 24 h	1.31E-03	1.545
inflammatory response (GO:0006954)	HSB 24 h	3.20E-06	1.754
inflammatory response to wounding (GO:0090594)	HSB 24 h	2.34E-02	–
negative regulation of apoptotic process (GO:0043066)	HSB 24 h	–	1.676
negative regulation of cell differentiation (GO:0045596)	HSB 24 h	–	1.554
negative regulation of cytokine production (GO:0001818)	HSB 24 h	4.95E-02	–
negative regulation of programmed cell death (GO:0043069)	HSB 24 h	–	1.690
response to bacterium (GO:0009617)	HSB 24 h	–	1.642
response to biotic stimulus (GO:0009607)	HSB 24 h	–	1.672
response to cytokine (GO:0034097)	WB 1 h	–	1.750
	HSB 24 h	–	1.584
response to external biotic stimulus (GO:0043207)	HSB 24 h	–	1.691
response to interleukin-1 (GO:0070555)	HSB 24 h	4.95E-02	–
response to other organism (GO:0051707)	HSB 24 h	–	1.663
wound healing (GO:0042060)	HSB 24 h	3.16E-02	1.650

**Table 4:** Genes of interest detected as differentially expressed (FDR adjusted p-value < 0.05) in at least one pairwise comparison of white bass (WB) and hybrid striped bass (HSB) gill tissue 1 h, 4 h, and 24 h after infection with *Flavobacterium covae*. Values are the log fold-change (logFC) between WB or HSB at a given timepoint relative to the 0 h control. Bold values indicate the logFC was greater than or less than the threshold for up- (logFC > 1) or down-regulation (logFC < −1), respectively. A dash (" - ") indicates a feature was not identified as a DEG for a given comparison(s). The Feature ID corresponds to the sequence contig ID in the *de novo* WB transcriptome used for alignment and annotation.

Gene Name	WB			HSB		
Feature ID	1 h	4 h	24 h	1 h	4 h	24 h
C-C chemokine 1	–	–	–	**−1.41**	**−2.30**	**3.88**
GAZY01117715.1						
C—C chemokine 2	–	–	–	–	**2.69**	**3.99**
GAZY01108419.1						
C-C chemokine 2	–	–	–	–	–	**2.96**
GAZY01081164.1						
C-C motif chemokine 16-like	**1.11**	**1.72**	**2.06**	–	–	**2.66**
GAZY01084862.1						
C-C motif chemokine 19-like	–	–	–	–	–	**3.85**
GAZY01117487.1						
Complement C1r-A subcomponent-like	–	**2.81**	–	–	–	**3.88**
GAZY01151558.1						
C-X-C motif chemokine 13-like isoform X1	–	–	–	–	–	**−2.15**
GAZY01111163.1						
C-X-C motif chemokine 9-like	0.42	–	–	–	–	**1.19**
GAZY01143486.1						
Cytosolic phospholipase A2 zeta-like	–	**4.17**	**3.46**	–	–	**−3.80**
GAZY01055201.1						
Eosinophil peroxidase	**1.93**	**1.52**	**3.49**	–	–	**−4.02**
GAZY01018677.1						
Granulocyte colony-stimulating factor-like	–	**5.44**	–	–	–	**7.63**
GAZY01087755.1						
Haptoglobin-like	–	**3.37**	–	–	–	**6.71**
GAZY01128146.1						
Heat shock 70 kDa protein	**2.55**	–	–	–	–	**4.71**
GAZY01114057.1						
Hepcidin	–	**2.07**	–	–	–	**7.06**
GAZY01116470.1						
Hepcidin 2 variant 2	–	**2.50**	–	–	–	**7.91**
GAZY01094059.1						
Interferon alpha/beta receptor 1a isoform X1	0.72	–	0.76	–	–	**1.42**
GAZY01056551.1						
Interferon gamma receptor 1-like precursor	–	–	–	–	–	0.86
GAZY01162208.1						
Interferon gamma receptor 2 precursor	–	–	–	–	–	**1.14**
GAZY01174528.1						
Interferon-gamma 1	–	–	–	–	–	**1.60**
GAZY01109918.1						
Interferon-induced GTP-binding protein Mx-like	–	**3.82**	–	–	–	–
GAZY01156755.1						
Interleukin-1 beta	–	**4.14**	–	–	**−1.30**	**6.96**
GAZY01123787.1						
Interleukin-17C-like	–	**2.44**	–	–	–	**3.68**
GAZY01107428.1						
Interleukin-8	–	**2.85**	–	–	–	**6.17**
GAZY01111394.1						
Leptin a	–	**−3.94**	–	–	–	**5.93**
GAZY01025909.1						
L-rhamnose-binding lectin SML-like	–	−**1.13**	–	**−1.32**	**−2.37**	**−1.41**
GAZY01138201.1						
Myosin heavy chain	–	–	–	–	–	**2.41**
GAZY01111398.1						
Myosin light chain 5	–	–	–	–	–	**2.82**
GAZY01095928.1						
NADPH oxidase organizer 1-like	–	–	–	–	**−2.96**	**3.15**
GAZY01149659.1						
Ribosomal protein L13	**4.50**	**3.71**	**3.87**	–	–	**2.77**
GAZY01030703.1						
Suppressor of cytokine signaling 1	–	–	–	–	–	**1.26**
GAZY01138235.1						
Suppressor of cytokine signaling 3-like	–	–	–	−0.53	−0.82	**1.66**
GAZY01136752.1						
Suppressor of cytokine signaling 5-like	–	–	–	–	–	0.51
GAZY01149939.1						
Toll-like receptor 2	–	–	–	–	−0.57	–
GAZY01158589.1						
Toll-like receptor 3	–	–	–	−0.36	−0.42	**−1.36**
GAZY01167522.1						
Toll-like receptor 3 isoform X1	–	–	–	–	–	−0.86
GAZY01110955.1						
Toll-like receptor 8	–	–	–	–	–	−0.66
GAZY01172426.1						
Toll-like receptor 9	–	–	**−1.72**	–	–	–
GAZY01162343.1						
Tumor necrosis factor alpha	–	0.70	0.85	–	–	**2.23**
GAZY01127947.1						
Tumor necrosis factor ligand superfamily member 6-like	–	–	–	–	−0.51	–
GAZY01130770.1						
Viperin	–	**2.29**	–	–	–	–
GAZY01132614.1						

A general trend of immune-related GO term enrichment and differential gene expression whereby related terms and transcripts were exclusively detected among WB comparisons, exclusively in HSB at 24 h post-infection, or identified at earlier timepoints (1 h and/or 4 h post-infection) in WB and at 24 h post-infection in HSB was observed. As discussed in greater detail below, this trend is broadly suggestive of a cytokine-mediated immune response in WB that is mounted early in the presence of *F. covae* to support resistance, and, conversely, a delay of the immune response in HSB such that at 24 h the concurrent cellular processes are involved in mitigating the impact of infection rather than prevention.

## Discussion

Columnaris remains a prevalent and devastating pathogen in freshwater fisheries and aquaculture operations despite the considerable amount of research on topics including, but not limited to, bacterial strain variation [2,3], pathogenesis [27,28], interaction and effect on host organisms [4,5], impact to industries [29,30], and treatments [31,32] conducted over the last several decades. The body of research on the causative *Flavobacterium* spp. to date reflects the high variation and complexity of host-responses across taxa that is, in large part, due to the ubiquitous and opportunistic nature of this pathogen. As such, the development of effective prevention measures against and treatments for columnaris requires investigation into the nuances of each host, their environment, and feasible pathogen/disease monitoring approaches, rather than a “silver bullet” strategy. The variation in *F. covae* susceptibility between WB, SB, and their hybrid observed here (Fig. 1), by Fuller et al.,[7] and by Farmer et al. [8] further reflects this complexity.

To gain insight into the host immune response processes underlying this variation between WB and HSB, we examined gene expression in WB and HSB gill tissue at different timepoints following *F. covae* infection. Comparisons of GO enrichment analysis results and differential expression of associated gene transcripts highlighted multiple cellular functions directly related to immune system processes (Tables 3 and 4). Several immune-related GO terms and genes detected in WB at earlier points (1 h and/or 4 h) following *F. covae* infection were not detected or were down-regulated in HSB until 24 h post-infection, suggesting the WB immune response mounted during initial stages of *F. covae* infection is also elicited in HSB but is relatively delayed. Additional processes highlighted by GO terms and DEGs detected in WB at 4 h and/or 24 h post-infection further reflect an efficient immune response per their roles in mitigation of immune processes toward cellular homeostasis and host protection. Conversely, other enriched GO terms and DEGs detected in HSB at 24 h post-infection reflect an active infection and dysregulation of cellular processes, indicating that *F. covae* infection had already occurred by this time and an immune response was activated in these fish. The delay of immune response mechanisms in HSB to 24 h post-infection is likely a major contributor to the variation in susceptibility to *F. covae* observed in the present study as the incidence of mortality began in HSB at a similar timepoint. Putative pathways of the immune response mechanisms and associated gene transcripts that support *F. covae* resistance (WB) or vulnerability (HSB) are elaborated upon below, and many are candidates for further investigation in functional studies, screening for breeding programs, potential targets for modification, and biomarkers for evaluating immune responses following preventative treatments and/or vaccination (Tables 3 and 4).

### Cytokine-mediated immune response mechanisms

The enrichment and up-regulation patterns of several GO terms and gene transcripts indicate that *F. covae* infection elicits a cytokine-mediated immune response in WB and HSB and that this response is relatively delayed in HSB (Tables 3 and 4). Among the cytokines detected as up-regulated earlier in WB (i.e., at 1 h or 4 h post-infection) compared to HSB (up-regulated at 24 h post-infection), several have well-characterized roles in immune response mechanisms and include: interleukin-1 beta (IL-1β), interleukin-17C-like (IL-17C), tumor necrosis factor alpha (TNF-α), granulocyte colony-stimulating factor-like (G-CSF), interleukin 8 (IL-8), C—C motif chemokine 16-like (CCL16), and C-X-C motif chemokine 9-like (CXCL9) (Table 4). Pro-inflammatory IL-1β is an activator of a major immune signaling effector, nuclear factor-κB (NF-κB), and, among other functions, facilitates a prompt response to infection or injury via inflammation [33]. As such, increased expression of IL-1β earlier in the presence of a pathogen, such as that detected here, is presumably advantageous, and, moreover, the down-regulation of IL-1β in HSB at 4 h post-infection, disadvantageous. IL-17C has been found to activate chemokines, TNF-α, antimicrobial peptides (AMPs), IL-1β, and other cytokines in fishes via the NF-κB pathway [34]. G-CSF is involved in regulating neutrophils (leukocytes) and promotes trafficking of granulocytes to enhance their immunological function [35]. G-CSF may also have antibacterial properties and promote inflammation, as expression of a cloned G-CSF homologue in large yellow croaker (*Larimichthys crocea*), *Lc*GCSFb, was increased in the gill and other mucus tissues after *Vibrio alginolyticus* infection and *in vivo* administration up-regulated proinflammatory cytokines IL6 and TNF-α and transcription factor C/EBPβ [35]. TNF-α is another pro-inflammatory cytokine that has an important role in adaptive immunity via initiation of apoptosis and T-cell development and migration, among other processes, and has been found to be increased in expression in *F. columnare* -resistant (strain LV-359–01) channel catfish (*Ictalurus punctatus*) [36]. IL-8 mediates the inflammatory response and has been found to function as a neutrophil activator (chemoattractant) in mandarin fish (*Siniperca chuasti*) after *F. columnare* G_4_ infection, among other species [37]. CCL16 is thought to be constitutively expressed and is involved in the maturation of immune cells [38]. CXCL9 is one of the lesser studied chemokines of the CXCL9–11/CXCR3 axis; however, a reduction of CXCL9 transcription following amoebic gill disease was identified in resistant Atlantic salmon (*Salmo salar*) compared to non-resistant fish [39].

Additionally, several cytokine-encoding transcripts that were only detected in HSB have been associated with pathogen resistance (or susceptibility) in a variety of fishes and may provide further insight into their immune response mechanisms and/or dysfunction (Table 4). For example, tumor necrosis factor ligand superfamily member 6-like (TNFSF6, or FasL/CD95L) is a pleiotropic cytokine that maintains immune homeostasis and general innate immune function, including via activation of NF-κB and apoptotic response after recognition of target cells by cytotoxic T lymphocytes (CTLs) [40]. Further, administration of TNFSF6 (intramuscularly) to golden pompano (*Trachinotus ovatus*) has been shown to reduce *Edwardsiella tarda* bacterial load and *E. tarda* proliferation was identified across tissues, including the gill, in TNFSF6-knockdown fish [40]. Down-regulation of TNFSF6 in HSB at 4 h post-infection may be of interest for investigations into antibacterial properties of mucosal tissues in the presence of pathogen and as it relates to host susceptibility. Further, C—C chemokine 1 (CC1) has been linked to a host-activated inflammatory response that supported European sea bass (*Dicentrarchus labrax*) resistance to *Amyloodinium ocellatum* and has been implicated in the early immune response to pathogens in Nile tilapia (*Oreochromis niloticus*) [41,42]. As such, the down-regulation of CC1 in HSB at 1 h and 4 h post-infection prior to up-regulation at 24 h may merit further investigation into the role of this and related chemokines in HSB immune response (Table 4). C—C motif chemokine 19-like (CCL19) has been implicated in the migration of antigen presenting cells (APC) and lymphocytes, and the up-regulation in gill tissue at 24 h post-infection is consistent with the findings of Fu et al. [43], whereby channel catfish susceptible to *F. columnare* (strain BGFS-27) showed greater expression of CCL19a.1 and CCL19a.2 at 24 h than at 4 h. Further, similar up-regulation patterns of one or more CCL19 member (CCL19a.1, CCL19a.2, and CCL19b) have been detected in other fishes after infection to various pathogens including channel catfish in response to *Edwardsiella ictaluri*, largemouth bass (*Micropterus salmoides*) following *Nocardia seriolae* infection, gray mullet (*Mugil cephalus*) following *Lactococcus garvieae*, orange-spotted grouper (*Epinephelus coioides*) following *Vibrio harveyi*, and koi carp (*Cyprinus carpio*) following *Aeromonas sobria* [43,44]. CCL19 was also one of the core transcripts associated to the GO terms cell chemotaxis and chemotaxis enriched as part of the host response of HSB at 24 h post-infection (Table 3), and therefore possibly reflecting immune cell circulation to the site of infection.

Notably, higher expression of interferon gamma (IFN‐γ), interferon gamma receptor 1-like precursor (IFNGR1), and interferon gamma receptor 2 precursor (IFNGR2) were also only detected in HSB at 24 h post-infection, perhaps suggestive of gene feedback regulation and/or crosstalk between immune processes elicited by IFN‐γ and other cytokines, including IL-1β and TNF-α, occurring in HSB at this timepoint in response to *F. covae*. IFN‐γ is well-characterized interferon typically produced by activated T-cells and natural killer (NK) cells in response to infection that also acts as a stimulator of the Janus kinase (JAK) and signal transducer and activator of transcription (STAT) pathway (JAK/STAT) implicated in growth, pathogen resistance, survival, differentiation, and the activation of downstream regulation of cytokines involved in immune response, among other processes [45]. Interestingly, expression of suppressor of cytokine signaling 1 (SOCS1), SOCS3, and SOCS5, negative regulators of JAK/STAT signaling [45], were detected as higher in HSB at 24 h relative to the 0 h control group. This may indicate that crosstalk between IFN‐γ, other cytokines, and associated pathways does not predominantly include JAK/STAT signaling, although this is outside of the scope of the current study.

It is important to note that excessive expression of pro-inflammatory cytokines, such as many of those named above, has been linked to host tissue damage [33]. Emam et al. [46], for example, posited that significant up-regulation of IL-1β detected in more-severely damaged Atlantic salmon (*Salmo salar*) may be related to a degree of tissue damage. Assuming the HSB in the current study underwent similar gill tissue damage to that observed in HSB 24 h after *F. covae* infection by Fuller et al. [7], the up-regulation of pro-inflammatory cytokines in HSB at 24 h may be indicative of the extent of gill tissue damage. However, it is presently unclear whether this link would be in addition to or in lieu of a cytokine-mediated immune response mounted later in HSB compared to WB and the high incidence of HSB mortality after *F. covae* exposure poses limitations to conducting research in this area.

### Differences in immune system modulation strategies and responses

Beyond those associated with a cytokine-mediated immune response, several other markers of an efficient and effective immune response in WB that generally follow the trend indicative of a delayed immune response in HSB were identified (Tables 3 and 4). This includes the up-regulation of complement C1r-A subcomponent-like (C1r-A), a member of the complement system, a conserved part of the innate immune system in vertebrates [47], and heat shock 70 kDa protein (HSP70), found to increase in the gill of *F. columnare* infected (strain BGFS-27) channel catfish and posited to be involved in limiting cellular damage and cellular detoxification [45]. Hepcidin (HAMP) and Hepcidin 2 (HAMP2) also followed this regulation pattern; the coordination between the two AMPs in iron homeostasis (HAMP) and innate antimicrobial immunity (HAMP2) activities to reduce iron availability to invading pathogens and promote cytokines has been characterized in HSB and other fishes [48], [49], [50]. Briefly, pathogen virulence, including that of *F. covae*, requires ferric iron uptake, and systemic iron levels positively regulate hepcidin synthesis to bind and degrade ferroportin after internalization into the cell, and subsequently prevent the release of additional iron into the bloodstream [50,51]. Moreover, RNA transport and rRNA binding enrichment across all WB comparisons and in HSB 24 h post-infection may reflect enhanced protein synthesis facilitating the observed differences in immune responsiveness [52]. This aligns with up-regulation patterns of ribosomal protein L13 (RPL13), a component of the large ribosomal subunit the proper functioning of which is also associated with greater protein synthesis and that was detected as overrepresented through Fisher's Exact Test by up-regulated DEGs in WB at 1 h post-infection (**Supplemental Table 3**) and enriched through GSEA by DEGs in WB at 1 h, 4 h, and 24 h and HSB at 24 h post-infection (**Supplemental Table 4**).

The differing regulation and exclusive detection of several GO terms and DEGs in WB or HSB comparisons also provides insight into key differences in the immune mechanisms of these fish (Tables 3 and 4). Of particular interest, leptin a (LEPA) was down-regulated in WB at 4 h and up-regulated in HSB at 24 h. Leptin is a hormone/cytokine with critical regulatory roles in growth, stress, immune function, and energy metabolism across vertebrate taxa, and knockdown of leptin has been shown to reduce the innate immune system and ultimately survivability of zebrafish (*Danio rerio*) in the presence of gram-negative *Pseudomonas aeruginosa* due increased bacterial load [53]. Investigating the differential regulation of LEPA in the WB and HSB in response to *F. covae* and other pathogens may be a key focus of future research toward understanding the differences in immune response modulation strategies between these fish due to its pleiotropic nature. Further, viperin (VIP; virus inhibitory protein, endoplasmic reticulum-associated, interferon-inducible) was only detected in WB at 4 h post-infection, VIP is classically characterized as involved in the innate immunity against viruses, however, its antibacterial properties have been suggested in other species as of late [54]. Further, GO terms associated with antioxidant activity and detoxification were identified in WB at 24 h and may represent the resolution of immune and inflammatory processes to prevent or mitigate tissue damage, counteract oxidative stress, and/or clearing pathogen-induced toxins. Conversely, several GO terms associated with wounding (e.g., blood coagulation, wound healing) and dysregulation of cellular processes (e.g., negative regulation of apoptosis and cell differentiation) were identified among the top for HSB at 24 h post-infection in parallel with many of the immune-related pathways and DEGs previously mentioned and coinciding with the onset of mortality of HSB after *F. covae* infection.

## Conclusions

The gill is a primary interface between fish and their environment, and therefore pathogens, and proper signaling and downstream mechanisms are critical for mounting a prompt and effective immune response. Here we present the first transcriptomic characterization of the gill immune response in WB and HSB after *F. covae* infection. Several pathways and genes of interest have been identified through this analysis and contribute to the understanding of mechanisms underlying the observed differences in resistance (WB) and susceptibility (HSB) of these fish. Among the immune-related GO terms and differentially expressed genes, those related to or encoding cytokines were predominant and detected as enriched and up-regulated in WB at early timepoints after infection (1 h and/or 4 h) indicative of an efficient, cytokine-mediated immune response that is ultimately effective towards protecting these fish. Conversely, many of the same gene transcripts and terms were not detected or were down-regulated in HSB until 24 h post-infection suggestive of a delayed immune response occurring around the onset of mortality in these fish and that is therefore insufficient. These and other findings can serve as a basis for future functional research investigating the nuanced differences between the WB and HSB immune response, and ultimately potential selective breeding and/or biotechnological intervention to support the production of fish with greater resistance to this ubiquitous and devastating pathogen.

## Funding sources

This project was supported by funds appropriated to the United States Department of Agriculture (ARS Research Projects #6028–31630–009–000-D and #6010–32000–027–000-D). Mention of trade names or commercial products in this article is solely for the purpose of providing specific information and does not imply recommendation or endorsement by the U.S. Department of Agriculture. The USDA is an equal opportunity provider and employer.

## Data availability statement

The RNA-Seq data have been made available in the NCBI Gene Expression Omnibus (GEO) repository under accession number GSE246056. All other data that support the findings of this study have been included in the manuscript and supplementary materials.

## CRediT authorship contribution statement

**Linnea K. Andersen:** Writing – review & editing, Writing – original draft, Visualization, Validation, Investigation, Formal analysis, Data curation. **Jason W. Abernathy:** Writing – review & editing, Supervision, Project administration, Methodology, Investigation, Funding acquisition, Formal analysis, Data curation, Conceptualization. **Bradley D. Farmer:** Writing – review & editing, Validation, Methodology, Investigation, Formal analysis, Data curation, Conceptualization. **Miles D. Lange:** Writing – review & editing, Supervision, Methodology, Investigation, Formal analysis, Conceptualization. **Matthew E. McEntire:** Writing – review & editing, Project administration, Investigation, Formal analysis. **Steven D. Rawles:** Writing – review & editing, Project administration, Investigation, Conceptualization.

## Declaration of competing interest

The authors declare that they have no known competing financial interests or personal relationships that could have appeared to influence the work reported in this paper.

## Appendix Supplementary materials

Image, application 1

Image, application 2
