# Supplementary material for: Gene expression profiles of white bass (Morone chrysops) and hybrid striped bass (M. chrysops x M. saxatilis) gill tissue following Flavobacterium covae infection
Source: Comp Immunol Rep. 2024 Apr 19;6:200144. doi: 10.1016/j.cirep.2024.200144 (PMC11061238; doi:10.1016/j.cirep.2024.200144)
Supplement: Supplementary file 1 [file mmc1.docx]

Supplemental Material

**Supplemental Table 1.** Results and primers used for RNA-seq validation by RT-qPCR.

**Supplemental Table 2.** All DEGs determined through pairwise comparisons.

**Supplemental Table 3.** All overrepresented GO terms identified via Fisher’s Exact Test.

**Supplemental Table 4.** All enriched GO terms identified via Gene Set Enrichment Analysis.
